# Mapping of CD4^+^ T-cell epitopes in bovine leukemia virus from five cattle with differential susceptibilities to bovine leukemia virus disease progression

**DOI:** 10.1186/s12985-019-1259-9

**Published:** 2019-12-16

**Authors:** Lanlan Bai, Shin-nosuke Takeshima, Masaaki Sato, William C. Davis, Satoshi Wada, Junko Kohara, Yoko Aida

**Affiliations:** 1 Viral Infectious Diseases Unit, RIKEN, 2-1 Hirosawa, Wako, Saitama 351-0198 Japan; 2 Photonics Control Technology Team, RIKEN Center for Advanced Photonics, 2-1 Hirosawa, Wako, Saitama 351-0198 Japan; 3 Faculty of Human Life, Department of Food and Nutrition, Jumonji University, 2-1-28 Sugasawa, Niiza, Saitama 352-0017 Japan; 4 Monoclonal antibody center, Department of Veterinary Microbiology & Pathology, Washington State University, Pullman, WA 99164-7040 USA; 5 Animal Research Center, Hokkaido Research Organization, 5-39-1 Shintoku, Hokkaido, 081-0038 Japan; 6 Nakamura Laboratory, Baton Zone Program, RIKEN Cluster for Science, Technology and Innovation Hub, 2-1 Hirosawa, Wako, Saitama 351-0198 Japan

**Keywords:** Bovine leukemia virus, Bovine leukocyte antigen class II, CD4^+^ T-cell epitope mapping, Disease susceptibility, Proviral load

## Abstract

**Background:**

Bovine leukemia virus (BLV), which is closely related to human T-cell leukemia virus, is the etiological agent of enzootic bovine leukosis, a disease characterized by a highly prolonged course involving persistent lymphocytosis and B-cell lymphoma. The bovine major histocompatibility complex class II region plays a key role in the subclinical progression of BLV infection. In this study, we aimed to evaluate the roles of CD4^+^ T-cell epitopes in disease progression in cattle.

**Methods:**

We examined five Japanese Black cattle, including three disease-susceptible animals, one disease-resistant animal, and one normal animal, classified according to genotyping of bovine leukocyte antigen (*BoLA*)-*DRB3* and *BoLA-DQA1* alleles using polymerase chain reaction sequence-based typing methods. All cattle were inoculated with BLV-infected blood collected from BLV experimentally infected cattle and then subjected to CD4^+^ T-cell epitope mapping by cell proliferation assays.

**Results:**

Five Japanese Black cattle were successfully infected with BLV, and CD4^+^ T-cell epitope mapping was then conducted. Disease-resistant and normal cattle showed low and moderate proviral loads and harbored six or five types of CD4^+^ T-cell epitopes, respectively. In contrast, the one of three disease-susceptible cattle with the highest proviral load did not harbor CD4^+^ T-cell epitopes, and two of three other cattle with high proviral loads each had only one epitope. Thus, the CD4^+^ T-cell epitope repertoire was less frequent in disease-susceptible cattle than in other cattle.

**Conclusion:**

Although only a few cattle were included in this study, our results showed that CD4^+^ T-cell epitopes may be associated with *BoLA-DRB3*-*DQA1* haplotypes, which conferred differential susceptibilities to BLV proviral loads. These CD4^+^ T-cell epitopes could be useful for the design of anti-BLV vaccines targeting disease-susceptible Japanese Black cattle. Further studies of CD4^+^ T-cell epitopes in other breeds and using larger numbers of cattle with differential susceptibilities are required to confirm these findings.

## Background

Bovine leukemia virus (BLV) is closely related to human T-cell leukemia virus types 1 and 2, and is associated with enzootic bovine leukosis, a common neoplastic disease in cattle [1, 2]. BLV infection can remain clinically silent, with cattle in a leukemic state, or can emerge as persistent lymphocytosis characterized by an increased number of B lymphocytes or rarely as B-cell lymphoma in various lymph nodes after a long period of latency [1, 2].

BLV contains the structural genes *gag*, *pol*, and *env* and the two regulatory genes *tax* and *rex*. The *gag* gene encodes three mature proteins, i.e., p15 (matrix protein), p24 (an abundant capsid protein), and p12 (nucleocapsid protein). The *tax* gene encodes Tax protein, which activates the transcription of BLV through the 5′ long terminal repeats of BLV [1, 3]. The BLV *env* gene encodes a mature surface glycoprotein (gp51) and a transmembrane protein (gp30). The gp51 protein is thought to be the major target of humoral immunity. Callebaut et al. [4] performed CD4^+^ T-cell epitope mapping of the gp51 protein and identified three epitopes: peptide 98–117, peptide 169–188, and peptide 177–192. Gatei et al. [5] also conducted epitope mapping in sheep, cows, and calves. They found two other gp51 CD4^+^ T-cell epitopes: peptide 51–70 and peptide 61–80. Mager et al. [6] performed a CD4^+^ T-cell proliferation assay using eight BLV-seropositive cows and found two epitopes in the p24 amino acid sequence: peptide 31–55 and peptide 141–165. Sakakibara et al. identified the T-cell epitopes Tax peptide 131–150 and Tax peptide 111–130, both of which contained epitopes recognized by T-cells from BALB/c and C57BL/6 mice, within the Tax protein [7]. However, to date, no Tax protein epitope mapping has been conducted in cattle. In fact, only two proteins, gp51 and p24, have been studied as CD4^+^ T-cell epitopes using the natural host of BLV.

BLV disease progression and proviral load are strongly related to major histocompatibility complex (MHC) class II alleles. The bovine MHC region is referred to as the bovine leukocyte antigen (*BoLA*) region [8, 9]. The *BoLA* class II region is divided into two distinct subregions: class IIa and class IIb. Class IIa contains classical class II genes, including at least two *DQA* genes, two *DQB* genes, one functional *DRB3* gene, and one *DRA* gene, and class IIb contains nonclassical class II genes. These class II genes encode proteins that are able to bind to the processed peptides and present the peptides to CD4^+^ T-cells. Class II molecules are formed by α- and β-chains encoded by distinct genes within the MHC region. For example, the α1 and β1 domains form the peptide binding groove [10]. MHC genes are highly polymorphic; to date, 65 *BoLA-DQA*, 87 *BoLA-DQB*, and 303 *BoLA-DRB3* alleles have been identified according to the BoLA Nomenclature Committee of the Immuno Polymorphism Database MHC database (http://www.ebi.ac.uk/ipd/mhc/bola). Therefore, class II molecules encoding distinct alleles may exert different effects on responses of T-cells via binding to different peptides directly within the peptide binding groove of the various class II molecules. Indeed, *BoLA-DRB3* polymorphisms are known to be associated with BLV-induced persistent lymphocytosis [11, 12] and BLV proviral load [13–15]. Recently, Miyasaka et al. reported that the *BoLA* class II allele DRB3*1601 was associated with a high BLV proviral load in Japanese Black cattle and that DRB3*0902 and DRB3*1101 were associated with a low proviral load [16]. Additionally, BoLA-DQA1*0204 and BoLADQA1*10012 were reported to be associated with low and high proviral loads, respectively [16]. Therefore, it is a hypothesis that disease-susceptible cattle may have fewer epitopes than resistant cattle, resulting in weak immune responses. Although several groups have used mice, sheep, and cattle to try to identify BLV epitopes recognized by CD4^+^ and CD8^+^ T-cells and B cells [4, 5, 7, 17–21], none of these studies have evaluated the roles of MHC polymorphisms.

Accordingly, in this study, we aimed to examine the roles of these polymorphisms and to map CD4^+^ T-cell epitopes in a preliminary study in BLV-susceptible and -resistant cattle infected with BLV.

## Methods

### Experimental infection with BLV and collection of blood samples

Five 5-month-old Japanese Black cattle (S2, S4, S6, R1, and N1), each of which was genotyped for *BoLA-DRB3* and *-DQA1* alleles using a polymerase chain reaction (PCR) sequence-based typing (SBT) method [22, 23], were experimentally challenged by intravenous injection of white blood cells obtained from BLV-seropositive Holstein-Friesian cattle (Table 1). The inoculated blood had 4 × 10^7^ copies of provirus, as estimated by BLV-CoCoMo-qPCR-2, a quantitative real-time PCR method that uses coordination of common motifs (CoCoMo) primers to measure the proviral loads of known and novel BLV variants in BLV-infected animals [24–27]. Blood samples were collected for approximately 5 months after the first inoculation, and DNA and serum samples were obtained.

**Table 1:** Blood samples used for epitope mapping

Animal no.	*BoLA-DRB3* allele^a^	*BoLA-DQA1* allele^a^	Breed	Age^b^ (months)	PBLs^c^	Proviral load	Anti-BLV antibody (ELISA)^e^
	A:B	A:B			(cells/μl)	(CoCoMo-qPCR)^d^	
Susceptible cattle							
S4	*1601:1601*	*10012:12021*	JB^f^	9	10,400	76,029	+
S2	*1601:1601*	*10012:10012*	JB	10	11,600	96,232	+
S6	*1601:1601*	*10012:10012*	JB	9	10,200	100,377	+
Resistant cattle							
R1	*2703:1501*	*10011:0204*	JB	9	4210	263	+
Normal cattle							
N1	*0503:1501*	*10011:0101*	JB	9	10,200	50,877	+

^a^Both alleles, A and B, for each animal are shown

^b^Age at the time of blood sample collection

^c^Peripheral blood lymphocyte (PBL) count per 1 μl of blood (measured at a single time point)

^d^Proviral load (expressed as the number of copies of provirus per 10^5^ peripheral blood mononuclear cells) was evaluated using the CoCoMo-qPCR-2 assay

^e^Enzyme-linked immunosorbent assay was performed using the BLV ELISA kit. +, BLV-positive

^f^JB, Japanese black

The study was approved by the Animal Ethical Committee and the Animal Care and Use Committee of the Animal Research Center, Hokkaido Research Organization (approval number 1302).

### Identification of BoLA-DRB3 and -DQA1 by PCR-SBT

*BoLA-DRB3* alleles were genotyped using the PCR-SBT method as previously described [22]. Briefly, *BoLA-DRB3* exon 2 was amplified by single-step PCR using the primer set DRB3FRW (5′-CGCTCCTGTGAYCAGATCTATCC-3′) and DRB3REV (5′-CACCCCCGCGCTCACC-3′), and the nucleotide sequences were determined. Sequence data were analyzed using ASSIGN 400 ATF software (Conexio Genomics, Fremantle, Australia), and both *BoLA-DRB3* alleles were determined.

*BoLA-DQA1* alleles were genotyped using the PCR-SBT method as previously described [23]. Briefly, nested PCR was performed using the primer pair DQA1intL2 and DQA1-677R for the first round of amplification and the primer pair DQA1intL3 and DQA1ex2REV2.1 for the second round. After amplicon purification using an ExoSAP-IT PCR product purification kit (Affymetrix, Cleveland, OH, USA), sequence processing and data analysis were performed as described for *BoLA-DRB3* typing.

### Preparation of peripheral blood mononuclear cells (PBMCs) and CD4^+^ T lymphocytes

PBMCs were separated according to the method of Miyasaka and Trnka [28], and CD4^+^ T-cells were purified using the MACS System (Miltenyi Biotech, Inc., Auburn, CA, USA). Briefly, PBMCs were incubated with the ILA11A monoclonal antibody (mouse anti-bovine CD4; VMRD, Inc., Pullman, WA, USA) and captured with anti-mouse IgG monoclonal antibodies conjugated to magnetic beads. Magnetic bead-bound cells were then separated on a MACS LS column (Miltenyi Biotech, Inc.). The purity of CD4^+^ T-cells was 85–89%.

### Synthetic peptides

A series of 20-mer peptides, each overlapping by 10 amino acids, was synthesized based on the reported sequences of BLV Gag (GenBank accession no. LC057268), Env (GenBank accession no. EF600696), and Tax (GenBank accession no. EF600696) proteins and purified using high-performance liquid chromatography to greater than 70% purity (Sigma, St. Louis, MO, USA). The peptides were then resuspended in 80% dimethyl sulfoxide (DMSO) to form stock solutions (2 mM), separated into aliquots, and stored at − 20 °C.

### Proliferation assay

Antigen-presenting cells (APCs) were prepared by treating PBMCs with 50 μg/mL mitomycin C (Sigma-Aldrich, St. Louis, MO, USA) in RPMI 1640 for 60 min at 37 °C. After washing five times in phosphate-buffered saline, cells were resuspended in RPMI 1640 and used as APCs. APCs (8 × 10^6^ cells/mL) and CD4^+^ T-cells (2 × 10^6^ cells/mL) were co-incubated in flat-bottomed 96-well microplates (Sigma-Aldrich, Trasadingen, Switzerland) in the presence of either 20 μM peptide or 0.8% DMSO (negative control) in a total volume of 110 μL in cell medium. The microplates were then incubated in a 5% CO_2_ humidified atmosphere at 37 °C. After 109 h of incubation, 10 μL Cell Counting Kit-8 solution (Dojindo Molecular Technologies, Kumamoto, Japan) was added to each well, and the microplates were incubated for an additional 4 h under the same conditions. The microplates were then read at an optical density of 450 nm. All test conditions were set up in triplicate. The measured absorbance was compared with that of control wells incubated without peptides, and the stimulation index (SI) was calculated using the following equation:

$$ \mathrm{Stimulation}\ \mathrm{Index}\ \left(\mathrm{SI}\right)=\frac{\left[\mathrm{PBMC},\mathrm{CD}4,\mathrm{peptide}\right]-\left[\mathrm{Medium}\ \mathrm{only}\right]}{\left[\mathrm{PBMC},\mathrm{CD}4,\mathrm{DMSO}\right]-\left[\mathrm{Medium}\ \mathrm{only}\right]} $$

### Measurement of BLV proviral load

BLV proviral loads in BLV-infected Japanese Black cattle were measured at a single time point using the BLV-CoCoMo-qPCR-2 method, as previously described [24–27, 29–31].

### Detection of anti-BLV antibodies in serum samples

An anti-BLV antibody enzyme-linked immunosorbent assay kit was used to detect antibodies according to the manufacturer’s instructions (JNC Corporation, Tokyo, Japan).

### Statistical analysis

The SI data were analyzed using *F*-tests and *t*-tests with the function program in Microsoft Excel. Results with *p* values of less than 0.01 were considered statistically significant.

## Results

### Genotyping of BoLA class II haplotypes and experimental challenge of five Japanese black cattle with BLV

*BoLA* class II genotypes are major regulators of BLV-induced persistent lymphocytosis progression and the dynamics of the provirus in the blood [11–14, 16, 32]. Although the MHC class II genotype is the most important factor that determines CD4^+^ T-cell epitopes, no studies have combined genotyping of *BoLA* alleles with epitope mapping. Here, we evaluated five *BoLA* class II-genotyped Japanese Black cattle (Table 1). Three (S2, S4, and S6) of five cattle were disease-susceptible cattle with the *BoLA class II* genotype which is associated with a high proviral load [16]. Two of these three cattle were homozygous for DRB3*1601 and BoLA-DQA1*10012, which are associated with a high proviral load [16], and one was homozygous for DRB3*1601 and heterozygous for BoLA-DQA1*10012. In contrast, the resistant animal (R1) carried the BoLA-DQA1*0204 allele, which is related to a low proviral load [16], and the normal animal (N1) did not harbor the known *BoLA-DRB3* or *BoLA-DQA1* alleles, which are associated with BLV proviral load. BLV provirus levels were markedly higher in all three susceptible cattle (S2, S4, and S6); however, levels were low and moderate in one resistant animal (R1) and one normal animal (N1), respectively (Table 1). These five cattle were experimentally infected with BLV and then used for CD4^+^ T-cell epitope mapping experiments.

### Proliferation of CD4^+^ T-cells isolated from BLV-infected cattle

**Fig. 1:**
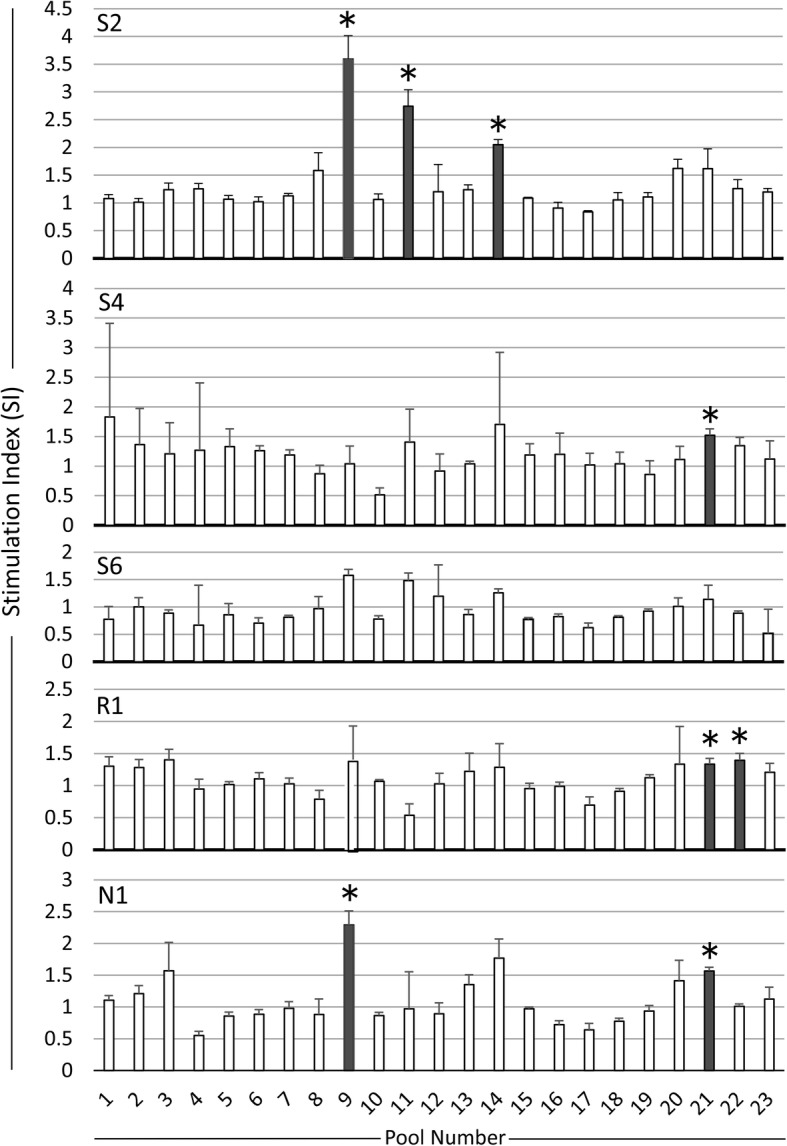
CD4^+^ T-cell proliferative responses to 23 peptide pools. PBMCs were obtained from five BLV-infected cattle (S2, S4, S6, R1, and N1). CD4^+^ T-cells were then isolated and used as effector cells. PBMCs were pre-treated with mitomycin C (4 × 10^5^/50 μl; 50 μg/ml) for 1 h at 37 °C and then co-incubated with CD4^+^ T-cells (1 × 10^5^/50 μl) and different peptide pools (each pool contained five different peptides, each at 20 μM) for 113 h at 37 °C. Cell Counting Kit-8 was used to measure CD4^+^ T-cell proliferation. The absorbance of the test wells was compared with that of control wells that did not contain peptides. The Stimulation Index (SI) was calculated as follows: $\mathrm{Stimulation}\ \mathrm{Index}\ \left(\mathrm{SI}\right)=\frac{\left[\mathrm{PBMC},\mathrm{CD}4,\mathrm{peptide}\right]-\left[\mathrm{Medium}\ \mathrm{only}\right]}{\left[\mathrm{PBMC},\mathrm{CD}4,\mathrm{DMSO}\right]-\left[\mathrm{Medium}\ \mathrm{only}\right]}$. The bars represent the mean ± standard deviation (SD) of triplicate wells. Asterisk and shade box bar mean the pool showed significantly higher value than DMSO (negative control) well (*p* < 0.01)

The synthesized peptides were grouped into 23 pools, each containing five peptides at a final concentration of 20 μM per peptide. At the first screening, CD4^+^ T-cells isolated from the five cattle were stimulated with each peptide pool, and proliferation was measured. No peptide pools significantly induced the proliferation of CD4^+^ T-cells from the susceptible animal S6 (*p* < 0.01). Peptide pools 9, 11, and 14 induced significantly high levels of proliferation in CD4^+^ T-cells from S2; pool 21 induced significantly high level of proliferation in cells from S4; pools 9 and 21 induced high levels of proliferation in cells from N1; and pools 21 and 22 induced high levels of proliferation in cells from R1 (Fig. 1).

**Fig. 2:**
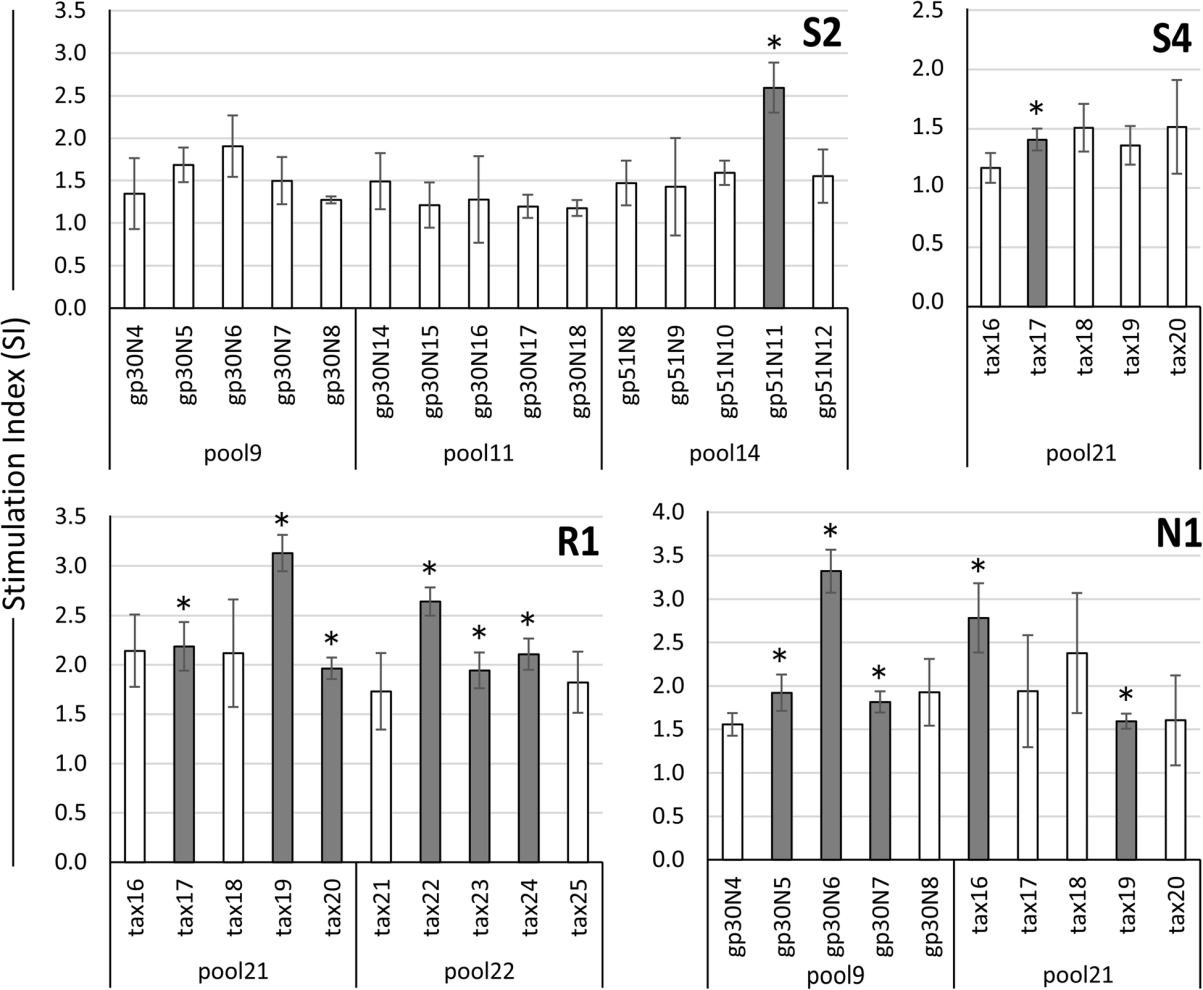
CD4^+^ T-cell proliferative responses to individual peptides within positive peptide pools. CD4^+^ T-cells (effector cells; 1 × 10^5^ cells/50 μl) from four BLV-infected cattle (S2, S4, R1, and N1) were co-incubated with mitomycin C-treated PBMCs (APCs; 4 × 10^5^ cells/50 μl) and incubated with either 80% DMSO (negative control) or peptide from pools 9, 11 and 14 (for S2), pool 21 (for S4), pools 20 and 21 (for R1), and pools 9 and 21 (for N1), all at a final concentration of 20 μM. The cells were incubated with peptide for 113 h at 37 °C and CD4^+^ T-cell proliferation was examined using Cell Counting Kit-8. The absorbance of the test wells was compared with that of control wells incubated without peptide and the Stimulation Index (SI) was calculated as follows: $\mathrm{Stimulation}\ \mathrm{Index}\ \left(\mathrm{SI}\right)=\frac{\left[\mathrm{PBMC},\mathrm{CD}4,\mathrm{peptide}\right]-\left[\mathrm{Medium}\ \mathrm{only}\right]}{\left[\mathrm{PBMC},\mathrm{CD}4,\mathrm{DMSO}\right]-\left[\mathrm{Medium}\ \mathrm{only}\right]}$. The bars represent the mean ± standard deviation (SD) of triplicate wells. Asterisk and shade box bar mean the peptide showed significantly higher value than DMSO (negative control) well (*p* < 0.01)

To further map the epitopes recognized by CD4^+^ T-cells from the five BLV-infected cattle, proliferative responses in the presence of peptide within the positive peptide pools were examined in proliferation assays. The peptides gp51N11 and tax17 induced particularly high levels of proliferation in CD4^+^ T-cells from S2 and S4, respectively. Five peptides (i.e., gp30N5, gp30N6, gp30N7, tax16, and tax19) induced high proliferation of CD4^+^ T-cells from N1, and six peptides (i.e., tax17, tax19, tax20, tax22, tax23, and tax24) induced high proliferation of CD4^+^ cells from R1 (Fig. 2).

### Overview of the positions of CD4^+^ T-cell epitopes identified in this study

**Fig. 3:**
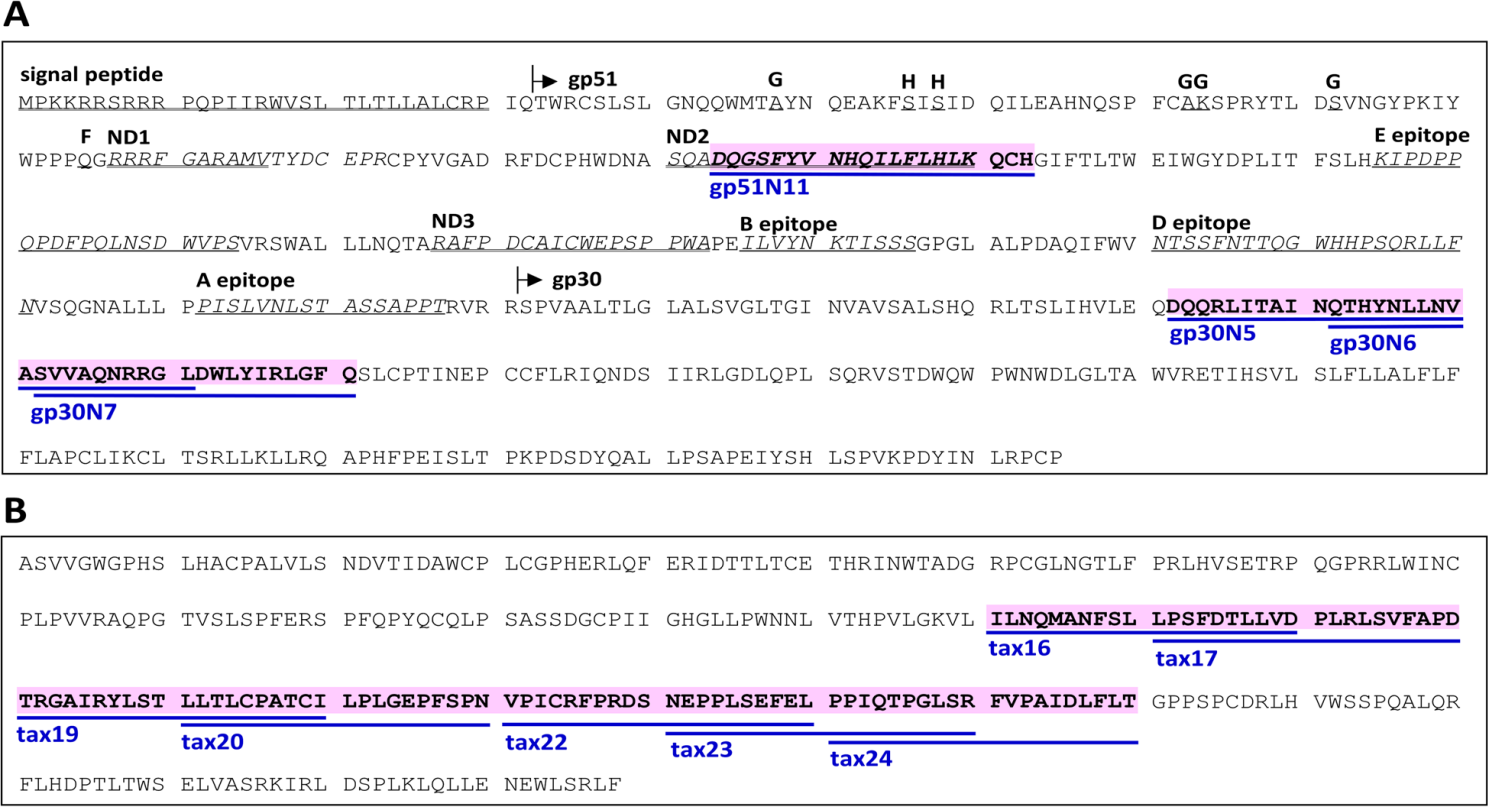
Schematic representation of CD4^+^ T-cell epitopes based on the deduced amino acid sequences of the BLV Env (gp51 and gp30) (**a**), and Tax (**b**) proteins. The epitope names, blue bold underline and pink denote the CD4^+^ T-cell epitopes identified in this study. The signal peptide is shown double under line. The putative gp51 and gp30 proteins are indicated with arrows. The three conformational epitopes (G, H and F) are shown under line. Five linear epitopes (A, B, C, D and E) are shown single under line and italic. Neutralization domains are shown in double under line and italic

In this study, we identified 11 types of 20-mer peptides that induced the proliferation of CD4^+^ T-cells collected from four of five BLV-infected cattle (Fig. 3). The number of CD4^+^ T-cell epitopes was positively related to proviral load, which dependent on the MHC class II genotype.

We identified a common epitope, gp30N6, recognized by CD4^+^ T cells from the normal animal (N1); this epitope corresponded to a putative immunosuppressive domain that affects the fusion activity of BLV in vitro [33] (Fig. 3). Moreover, gp30N5 and gp30N7, which were located on either side of gp30N6, were also recognized as CD4^+^ T-cell epitopes in N1. Although many tax peptides showed high SI values, these peptides were not identified as CD4^+^ T-cell epitopes because of the high standard errors observed during peptide screening (Fig. 2). The SI average of peptides from pool 21 tended to be high. Four peptides, i.e., tax20, tax22, tax23, and tax24, only induced proliferation in R1 and showed low proviral loads. In addition, N1 also had two peptides, i.e., tax16 and tax19, which were identified as CD4^+^ T-cell epitopes. Therefore, the tax extracellular domain was considered a common CD4^+^ T-cell epitope in this study.

Although few cattle were examined in this study, we found strong evidence that the genetic background may affect the selection of proteins as immune targets for CD4^+^ T cell-associated immune responses. Further studies using experimental infection should be performed to confirm our results.

## Discussion

In this study, we screened 115 synthetic peptides encompassing the Gag proteins (p15, p24, and p12), Env proteins (gp51 and gp30), and Tax proteins of BLV. From this preliminary study, we identified 11 epitopes recognized by CD4^+^ T-cells isolated from five cattle (S2, S4, S6, R1, and N1) showing differing susceptibilities to BLV according to *BoLA* class II haplotypes. This is the first study to use MHC class II-genotyped cattle to map CD4^+^ T-cell epitopes in BLV, and our result showed that CD4^+^ T-cell epitopes derived from disease-susceptible cattle harboring the BoLA-DRB3*1601 homozygous genotype (*n* = 3) were fewer in number than those in resistant (*n* = 1) and normal cattle (*n* = 1). The *BoLA-DRB3* gene regulates both antigen epitope recognition and the magnitude of antigen-specific T-cell responses mounted upon exposure to infection [8, 9]. Similarly, Nagaoka et al. [34] also showed the weak reactivity for BLV peptide vaccination in BLV-susceptible sheep and found that susceptible sheep developed BLV-induced lymphoma after challenge by BLV. These results suggested that immune responses contributed to individual differences in CD4^+^ T-cell epitopes owing to MHC class II polymorphisms.

Three BLV peptides, i.e., Env 98–117 [4], Env 51–70, and Env 61–80 [5], are known CD4^+^ T-cell epitopes. Here, we identified one CD4^+^ T-cell epitope within the gp51 protein, namely, gp51N11, and showed that 17 of the 20 amino acid sequences of gp51N11 were identical to Env 98–117. Peptide pool 14, which contained gp51N11, showed a relatively high SI, indicating that this region contained epitopes recognized by CD4^+^ T-cells. Sakakibara et al. identified T-cell epitopes within the Tax protein [7], i.e., peptide 131–150 (IGHGLLPWNNLVTHPVLGKV) and peptide 111–130 (SPFQPYQCQLPSASSDGC), which contained epitopes recognized by T-cells from BALB/c and C57BL/6 mice, respectively. These regions corresponded to tax11 and tax14, neither of which were identified as epitopes in the current study. These findings suggested that CD4^+^ T-cell epitopes are different in mice and cattle. Interestingly, tax17, tax19, tax20, and tax22–24 (detected in R1 in our study) corresponded to a leucine-rich region (tax157–197) that may be involved in heterologous protein interactions [35]. According to a previous study [16], the resistance alleles *BoLA-DRB3* and *BoLA-DQA* are commonly observed in Japanese Black and Holstein cattle, whereas susceptible alleles differed. Although there was only one resistant animal, more epitopes from Tax protein were identified in resistant cattle than in other cattle, suggesting that CD4^+^ T-cell epitopes (Tax22–24) from Tax protein may induce strong immune responses. Additional studies with more cattle are required to further confirm these findings.

## Conclusion

We successfully identified 11 BLV epitopes recognized by CD4^+^ T-cells from four of five cattle, including four types of *BoLA* class II haplotypes. Among CD4^+^ T-cell epitopes related to the MHC class II genotype, fewer CD4^+^ T-cell epitopes were observed in susceptible cattle than in resistant and normal cattle. Although few samples were evaluated, the result showed that antigens were restricted according to *BoLA* class II haplotype, indicating that genotyping is important for determining antigenic epitopes recognized by the host immune response.

## Data Availability

All data and materials are included in this published article.

## Abbreviations

APCs: Antigen-presenting cells
BLV: Bovine leukemia virus
BoLA: Bovine leukocyte antigen
CoCoMo: Coordination of common motifs
DMSO: Dimethyl sulfoxide
gp30: Transmembrane protein (gp30)
gp51: Surface glycoprotein (gp51)
MHC: Major histocompatibility complex
OD: Optical density
PBMC: Peripheral blood mononuclear cell
SBT: Sequence-based typing
SI: Stimulation Index
